# Intrapelvic Femoral Head Dislocation without Associated Proximal Femur Fracture: A Case Report and Description of Closed Reduction Technique

**DOI:** 10.1155/2019/1913673

**Published:** 2019-12-13

**Authors:** S. Craig Morris, Priyantha L. Wickramanayake, Peter J. Wilton, Jonathan L. Allen

**Affiliations:** ^1^Department of Orthopedic Surgery, Loma Linda University, Loma Linda, CA, USA; ^2^Department of Orthopedic Surgery, Arrowhead Regional Medical Center, Colton, CA, USA

## Abstract

Traumatic hip dislocations are potentially devastating injuries, especially in young patients, and require emergent orthopedic treatment. Given the significant amount of energy required to cause these injuries, a high index of suspicion is necessary to identify related injuries. The associated injuries, direction of dislocation, and time between injury and reduction represent the known prognostic factors, based on limited available research. Intrapelvic hip dislocations represent an uncommon variant of the traumatic hip dislocation, with all previously reported cases involving ipsilateral proximal femur fractures. We present a case of intrapelvic femoral head dislocation without an associated proximal femur fracture, as well as the maneuvers used to treat the patient via a closed reduction.

## 1. Introduction

Traumatic hip dislocations are a serious orthopedic injury with increasing incidence, typically related to high energy trauma, most commonly motor vehicle accidents [1]. Due to the risk of osteonecrosis of the femoral head, many authors recommend that these injuries should be reduced emergently, especially in younger patients [1]. Other potential complications include sciatic nerve injuries and the development of posttraumatic osteoarthritis [2]. Hip dislocations are classified as simple if there is no associated fracture of the proximal femur or acetabulum, or complex if there is associated fractures. These injuries are also defined based on the direction of dislocation of the femoral head relative to the acetabulum, posterior, or anterior. Early reports also described a central dislocation, but these are thought to be more accurately defined as acetabulum fractures as opposed to traumatic hip dislocations. More detailed classification schemes for dislocations and fracture dislocations have been described previously by Thompson and Epstein [3], Stewart and Milford [4], and Brav [5]. Anterior dislocations are less common and are typically subdivided based on the position of the femoral head on an AP pelvic radiograph: obturator, pubic, and iliac [1–3]. Another subtype of anterior dislocation less frequently used is the perineal type, which describes a dislocation where the femoral head is more inferior than in the standard obturator dislocation [6]. A common complication of anterior hip dislocations is impaction of the femoral head on the anteroinferior rim of the acetabulum as it exits the acetabulum [2, 7]. Unique anterior hip dislocations through or into the scrotum have been reported in the literature [8, 9]. The anterior obturator class of dislocations has been reported in the literature to have a variety of presentations, including with associated femoral neck fractures [10, 11], with associated femoral head fracture [12], and with intrapelvic dislocation of the proximal femur through the obturator foramen [13–15]. Intrapelvic dislocations, while rare, have been reported previously, though always with associated proximal femur fracture [13–17], or fracture of both the proximal femur and the acetabulum or the pelvic ring [18–20]. There have been no reported cases in the literature of intrapelvic hip dislocation without associated proximal femur fracture. After discussion, the patient signed informed consent regarding the use of his medical information in this case report.

## 2. Case Presentation

**Figure 1:**
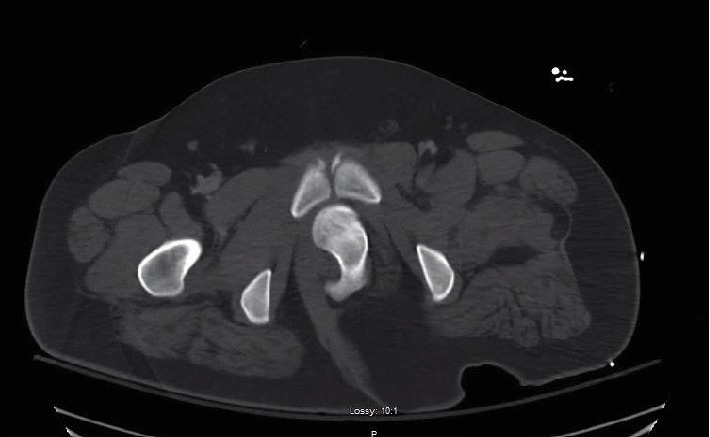
Axial slice of CT abdomen and pelvis with contrast demonstrating intrapelvic dislocation with the left femoral head just posterior to pubic symphysis.

**Figure 2:**
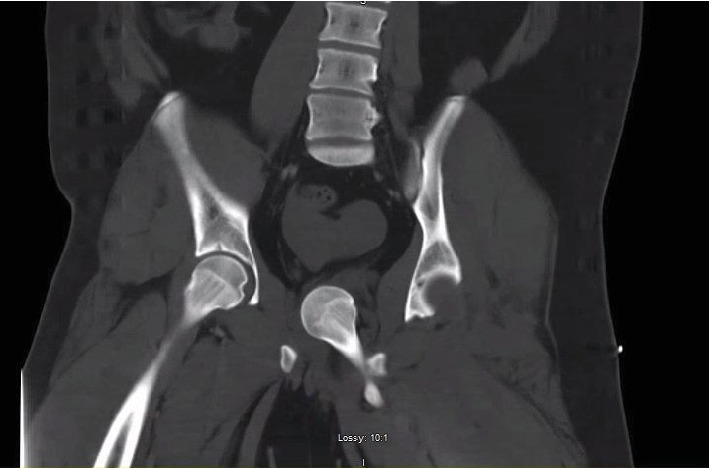
Coronal slice of CT abdomen and pelvis with contrast demonstrating medial displacement of femoral head into the true pelvis with empty acetabulum on the left.

An 18-year-old morbidly obese male with no reported past medical history presented to the emergency room after a high-speed motor vehicle collision (MVA). He had been positioned in the middle of the backseat and was ejected from the vehicle. The injury was reported to have occurred 1 hour prior to presentation at the hospital. Field responders had placed the patient on a backboard and placed a cervical collar. He was resuscitated with intravenous fluids during transport. No manipulation of the extremities was completed prior to arrival. Upon arrival to the emergency room, he was intubated and sedated for airway protection due to low Glasgow Coma Scale. He had sustained facial lacerations and closed head trauma. CT of the abdomen and pelvis was obtained showing a dislocated left hip, with the femoral head displaced into the intrapelvic space compressing the urethra (Figures 1 and 2). There was a small bony fragment noted, representing a minor acetabulum fracture, but no evidence of proximal femur fracture. He presented in critical condition due to subdural hemorrhage and was taken emergently to the operating room for a craniotomy performed by the neurosurgical team. Due to the acuity of his head injury, no other imaging of the orthopedic injuries was obtained prior to transfer to the operating room. He was already in the operating suite after completion of the craniotomy when the orthopedic team was able to evaluate the patient. Physical exam demonstrated gross deformity of bilateral lower extremities as both legs were externally rotated and shortened. On the right thigh, there was crepitus at the distal femur suggesting a distal femur fracture. Fluoroscopic imaging was obtained intraoperatively, confirming a right distal femur fracture and left hip dislocation. After receiving clearance from the trauma surgery and neurosurgery teams for further intervention on the patient at that time, the decision was made for emergent closed reduction of the left hip under general anesthesia given the intrapelvic dislocation of the femoral head in this young patient. Given the significant displacement of the femoral head, it was discussed that open reduction may be necessary, likely via a medial approach to the hip. Given the critical condition of the patient, closed reduction was preferred, if possible. The orthopedic surgery team began the closed reduction procedure approximately 3 hours and 30 minutes after presentation to the hospital and therefore approximately 4 hours and 30 minutes after injury.

**Figure 3:**
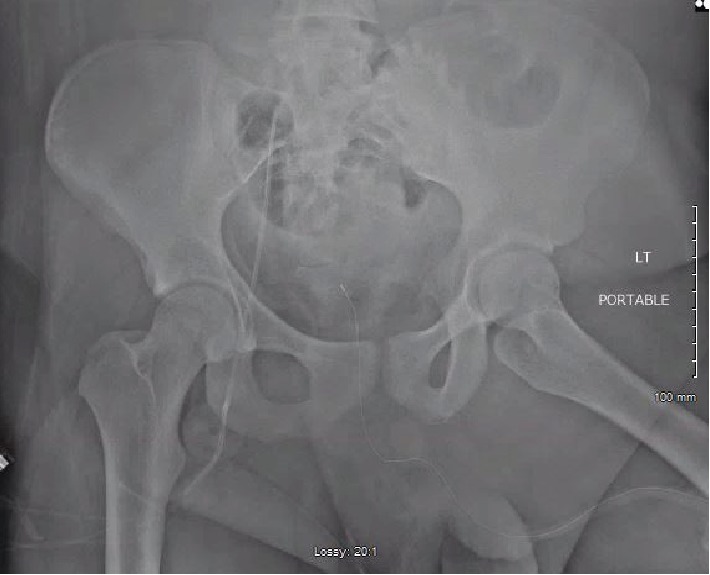
Intraoperative fluoroscopic AP pelvis demonstrating concentric reduction of the left femoral head.

Fluoroscopy was used to aid in the closed reduction. The primary surgeon began by applying gentle traction in line with the position of the left lower extremity. Due to his massive body habitus, more forceful traction was required, and a sheet was placed across his body at the level of iliac crests with an assistant pulling countertraction. Initial attempts at closed reduction using typical reduction techniques were ineffective given the position of the dislocated femoral head within the pelvis. After several minutes of traction, fluoroscopy revealed that the femoral head was still significantly displaced medially into the patient's intrapelvic region. At this time, additional countertraction, via another sheet, was placed around the left thigh and a laterally applied force was administered by another surgeon while the primary surgeon continued to apply longitudinal traction. Based off of the presumed injury pattern of hyperabduction in a frog leg position from being thrown forward into the front seats, hyperabduction and flexion combined with longitudinal traction were utilized to slowly mobilize the dislocation outside of the pelvis. Internal rotation was also used to manipulate the femoral head inferior to the ischium followed by external rotation to move the head out of the true pelvis. After significant time and effort, fluoroscopic images demonstrated that the hip was out of the pelvis and had effectively been converted to a more commonly encountered posterior dislocation. At this point, hyperabduction was no longer necessary. Instead, longitudinal traction, hip flexion, and external rotation were utilized until an audible clunk was appreciated, and a fluoroscopic image confirmed reduction of the left femoral head (Figure 3). Provisional treatment of the right distal femur fracture was then administered via skeletal traction to the right lower extremity through a tibial pin. The total operating time for the closed reduction as well as traction pin in the contralateral femur was approximately 1 hour and 15 minutes.

At this point, surgeons from other specialties took over care in the ongoing management of this polytraumatized patient and the urological surgery team was consulted to evaluate and manage any potential urethral injury from the dislocation. Placement of Foley in the trauma bay had been unsuccessful. After reduction of the hip, the urology team was able to place a Foley catheter without difficulty with return of normal-appearing urine without any evidence of hematuria. Given the ease of placement and benign appearance of the urine, no further imaging of the urinary tract was recommended by the urology service. The Foley catheter was removed later during hospitalization without complication.

**Figure 4:**
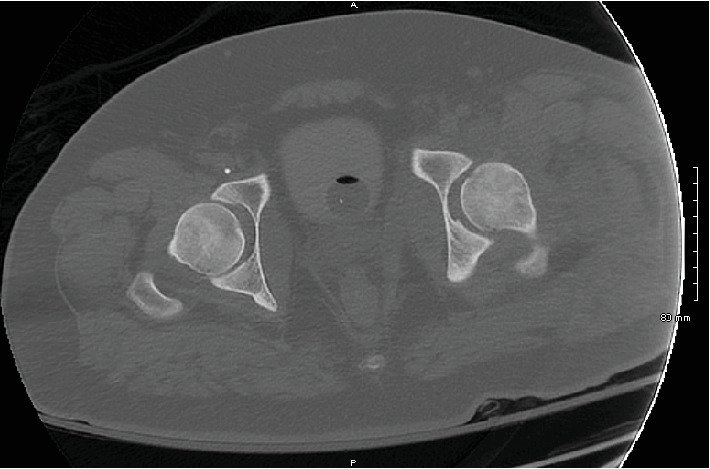
Postreduction axial slice of CT pelvis without contrast demonstrating concentric reduction of the left femoral head.

**Figure 5:**
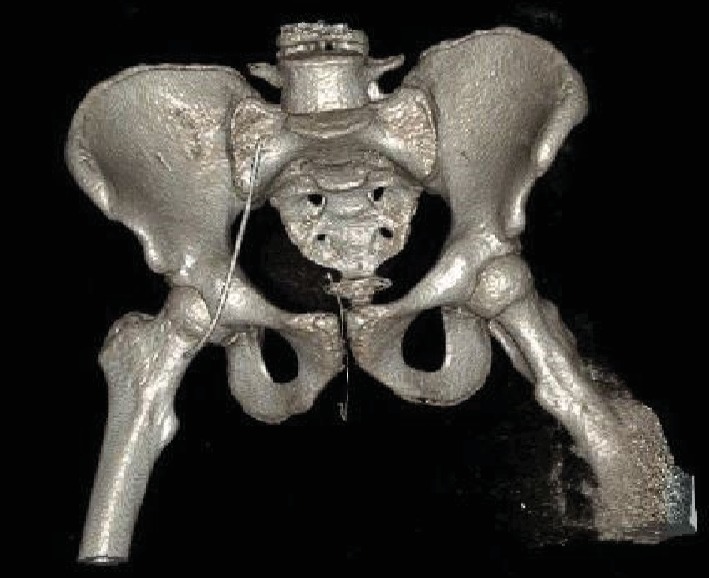
Postoperative 3D reconstruction from CT pelvis without contrast demonstrating reduced left femoral head without associated femoral fracture.

The patient was admitted to the surgical intensive care unit after leaving the operating room. Postoperative radiographs and CT scan showed reduction of the left hip (Figures 4–5). An 8 mm bony fragment from the acetabulum was again noted, which was presumed to represent an avulsion fracture from the ligamentum teres, but the scan demonstrated concentric reduction of the femoral head and no evidence of femoral fracture. Three days after presentation, the patient was weaned from sedating medications and extubated. His right lower extremity remained in skeletal traction until definitive treatment could be completed in the form of retrograde intramedullary nailing of the femur on hospital day 4, which was completed without complication. His mental status continued to improve, and he began to work with physical and occupational therapists during his admission. He was made weight bearing as tolerated on the right lower extremity but kept nonweight bearing on the left lower extremity. The patient was discharged to an acute inpatient rehab facility on hospital day 21.

**Figure 6:**
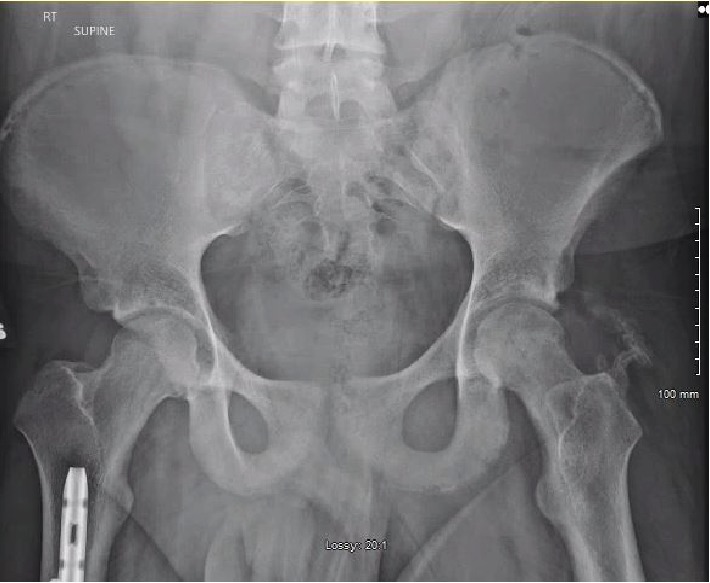
Follow-up AP pelvis radiograph at 9 weeks postinjury demonstrating concentric reduction of the left femoral head with heterotopic ossification without evidence of femoral head collapse.

At a follow-up visit to the orthopedic surgery clinic 9 weeks after injury, the patient reported occasional pain to the left hip and lower back after extended sitting in the wheelchair. He had continued in physical therapy as an outpatient and had been ambulating with a walker. His mental status had improved as he was now not only alert and oriented but also conversant throughout the exam. Physical exam demonstrated left hip range of motion of 0-60 degrees flexion (0-90 degrees flexion contralateral side) and 4/5 strength with hip flexion (4/5 strength hip flexion contralateral side). Distally, he was able to actively dorsiflex and plantarflex the ankle as well as flex and extend the great toe with 5/5 motor strength throughout bilateral lower extremities. Sensation was intact to light touch in superficial peroneal, deep peroneal, sural, saphenous, and plantar nerve distributions. He had 2+ dorsalis pedis and posterior tibial artery pulses. His gait was antalgic with a walker with decreased stance phase on the left leg. Radiographs showed concentric left hip with some irregularity noted in the femoral head concerning for osteonecrosis without femoral head collapse, as well as heterotopic ossification in surrounding soft tissues (Figure 6). At this point, he was made weight bearing as tolerated on the left lower extremity and continued in outpatient physical therapy.

**Figure 7:**
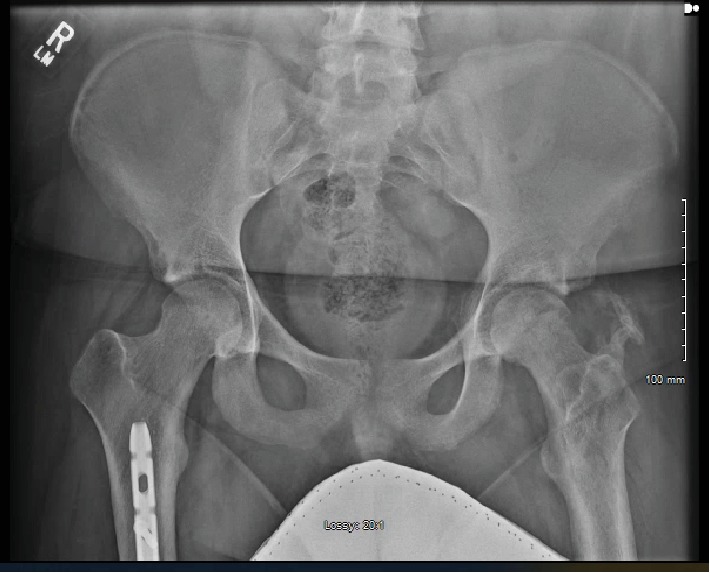
Follow-up AP pelvis radiograph at 4.5 months postinjury demonstrating further heterotopic ossification and some irregularity of the femoral head concerning for osteonecrosis.

**Figure 8:**
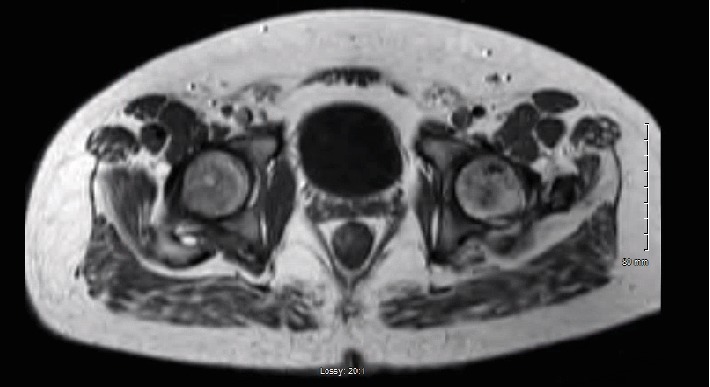
T1 axial image from MRI obtained 7 months postinjury demonstrating focal areas of hypointensity within the left femoral head consistent with osteonecrosis.

**Figure 9:**
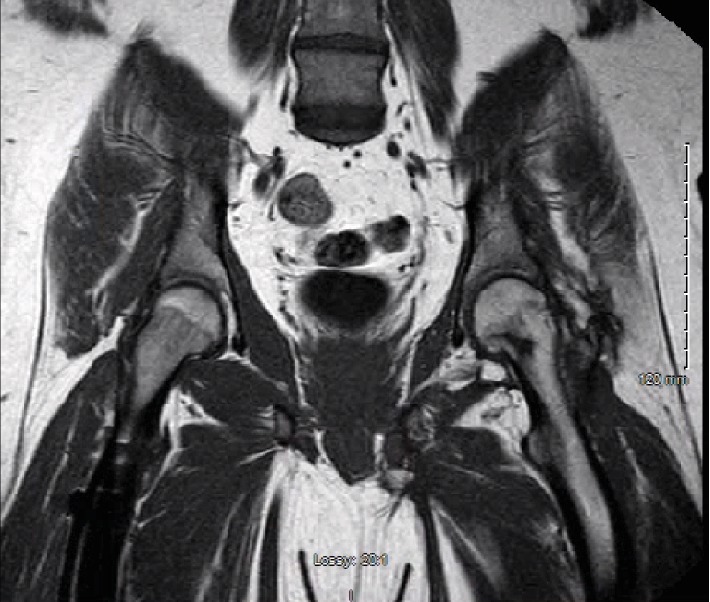
T1 coronal image from MRI obtained 7 months postinjury demonstrating focal areas of hypointensity within the left inferior femoral neck consistent with osteonecrosis.

The patient returned to follow-up in clinic 4.5 months after injury. At that time, he reported no left hip pain at rest. He had continued in physical therapy on an outpatient basis. Physical exam was notable for improved strength with hip flexion of 5/5 in bilateral lower extremities but otherwise was unchanged from prior exam. An AP pelvis X-ray was obtained which showed maturation of the heterotopic ossification adjacent to the left hip (Figure 7). Again noted was some irregularity in the femoral head but no evidence of femoral head collapse. An MRI of the pelvis was obtained 7 months postinjury, demonstrating focal areas of hypointensity on T1 images within the femoral head and inferior neck consistent with osteonecrosis, without evidence of femoral head collapse (Figures 8 and 9). Metal artifact from the right femur implant limited the ability of the MRI to evaluate the soft tissues in detail.

## 3. Discussion

Unfortunately, given the nature of the injury, the available literature on traumatic hip dislocations is largely limited to small, retrospective studies. In many of these cases, precise details regarding the time of injury to reduction and details of treatment are lacking given the retrospective nature. Moreover, results are conflicting as some have even reported good outcomes after reductions of chronic dislocations [21]. As such, conclusions regarding definitive treatment and prognosis are limited. Furthermore, given the unique displacement of the femoral head in our patient, it is unclear how the available literature will correspond to this patient's long-term prognosis. Based on available data, however, authors have reported that the most important factors in long-term prognosis for these injuries is the direction of the dislocation [22], time between injury and reduction [23], and the associated injuries [22, 23]. Associated injuries frequently occur with traumatic hip dislocations, involving up to 70.8% of patients [24], as was the case with our patient. Associated injuries can involve craniofacial and cervical spine fractures, closed head injuries, abdominal lacerations or organ injuries, lung contusions, and rib fractures, as well as fractures or dislocations of the upper and lower extremities [24, 25]. Due to the high energy required to produce a traumatic hip dislocation, a high suspicion for concomitant injuries must be maintained with thorough primary and secondary surveys completed according to advanced trauma life support protocols.

The presentation of our patient, with the femoral head located inside the pelvis without an associated proximal femur fracture likely resulted from a unique mechanism during the injury. It is suspected that the patient was thrown forward into the front seats prior to ejection from the vehicle. With a combination of hip flexion, hyperabduction, and internal fixation, the femoral head likely dislocated inferiorly from the acetabulum, then translated under the ischial tuberosity with subsequent external rotation bringing the femoral head superiorly up into the pelvis and ultimately resting behind the pubic symphysis. It is also likely that the patient's left leg became stuck momentarily during the ejection process resulting in the significant traction force required to clear the bony anatomy of the pelvis prior to dislocating medially. Given the strong sacrospinous and sacrotuberous ligaments, as well as lack of sacroiliac joint widening on imaging, it is less likely that the hip dislocated posteriorly and entered the pelvis from that direction. Significant energy and displacement, which resulted in the intrapelvic dislocation, likely caused significant injury to soft tissue structures, including but not limited to the ligamentum teres, hip capsule, short external rotators, and iliopsoas tendon. By visualizing the mechanism of injury and likely course of displacement into the pelvis, the operating surgeons were able to reduce the hip closed by reversing the suspected steps of dislocation. Reducing the hip with closed maneuvers was the ideal treatment for this polytraumatized patient who was still being resuscitated and treated for a traumatic brain injury. While the postoperative MRI demonstrates findings concerning for osteonecrosis, the patient has largely had a favorable outcome with no femoral head collapse, no hip pain at rest, and functional mobility.

Traumatic hip dislocations in young patients represent a true orthopedic emergency due to the potentially devastating complications including avascular necrosis and posttraumatic arthritis. Intrapelvic dislocations represent a very rare subset of these injuries and previously had only ever been reported with associated proximal femur fracture. This case demonstrates that not only can intrapelvic dislocations occur without femur fracture but that these injuries can be amenable to closed reduction despite their significant displacement into the true pelvis.
